# Elevated serum 4HNE plus decreased serum thioredoxin: Unique feature and implications for acute exacerbation of chronic obstructive pulmonary disease

**DOI:** 10.1371/journal.pone.0245810

**Published:** 2021-01-25

**Authors:** Jia Liu, Jin Huang, Hu Liu, Chang Chen, Jianying Xu, Liangwei Zhong

**Affiliations:** 1 Medical School, University of Chinese Academy of Sciences, Huai Rou, Beijing, China; 2 Respiratory Department, Shanxi Bethune Hospital/Shanxi Academy of Medical Sciences, Taiyuan, China; 3 National Laboratory of Biomacromolecules, Institute of Biophysics, Chinese Academy of Sciences, Chaoyang District, Beijing, China; Xiangtan University, CHINA

## Abstract

Acute exacerbation of chronic obstructive pulmonary disease (AECOPD) is a global problem with high mortality. Its pathogenesis is not fully understood. To reveal new serum feature of AECOPD and their potential implications, we have analyzed 180 serum samples, and found that in the serum of AECOPD patients, 4-hydroxy-2-nonenal (4HNE)-protein adducts are dynamically increased as partial pressure of oxygen (PaO_2_) drops, which is accompanied by progressively decreasing thioredoxin reductase (TrxR1) and thioredoxin (Trx1), as compared with those of healthy people. This phenomenon is unique, because acute hypoxia patients have 1.1-fold or 1.7-fold higher serum TrxR1 or Trx1 activity, respectively, than healthy people, in keeping with low 4HNE level. Moreover, serum 4HNE-protein adducts may form disulfide-linked complexes with high-molecular-weight, the amount of which is significantly increased during AECOPD. Serum 4HNE-protein adducts include 4HNE-Trx1 adduct and 4HNE-TrxR1 adduct, but only the former is significantly increased during AECOPD. Through cell biology, biochemistry and proteomics methods, we have demonstrated that extracellular 4HNE and 4HNE-Trx1 adduct affect human bronchial epithelial cells *via* different mechanisms. 4HNE-Trx1 adduct may significantly alter the expression of proteins involved mainly in RNA metabolism, but it has no effect on TrxR1/Trx1 expression and cell viability. On the other hand, low levels of 4HNE promote TrxR1/Trx1 expression and cell viability, while high levels of 4HNE inhibit TrxR1/Trx1 expression and cell viability, during which Trx1, at least in part, mediate the 4HNE action. Our data suggest that increasing serum 4HNE and decreasing serum Trx1 in AECOPD patients are closely related to the pathological processes of the disease. This finding also provides a new basis for AECOPD patients to use antioxidant drugs.

## Introduction

Chronic obstructive pulmonary disease (COPD) kills more than 3 million people worldwide every year [1]. This disease is characterized by airway airflow limitation and dysfunction in lungs and bronchial epithelium [2]. Acute exacerbation of COPD (AECOPD) has high morbidity and mortality, and the life quality of patients may be permanently impaired [3, 4]. The efficient prevention and treatment of AECOPD are currently hampered because the detailed pathophysiology remains largely unclear [5]. Chronic exposure to hypoxia markedly increased reactive oxygen species (ROS) levels in human pulmonary arterial smooth muscle cells [6]. Excessive ROS could cause oxidative stress. Worsening hypoxemia/the increased oxidative stress was considered a hallmark of AECOPD [7, 8]. In general, hypoxia can cause lung oxidative damage [9]. Oxidation of cell membrane phospholipids results in the elevation of various lipid hydroperoxides and aldehydic molecules, including 4-hydroxyl-2-nonenal (4HNE). Oxidative stress is increased further in AECOPD [10]. 4HNE is a key mediator of oxidant-induced cell signaling in lung inflammation of COPD patients [11].

In response to oxidative stress, 4HNE reaches a local concentration up to 5 mM [12], whereas under physiological conditions its concentration in human plasma is less than 1 μM [13]. With the features of low molecular weight, lipid-solubility and relative long life, 4HNE can cross cell membranes and diffuse from one place to another easily [13]. At high concentrations, 4HNE may cause cytotoxic effect through modifying Cys, Lys and His residues in proteins [14] to reduce their activities. *In vivo*, 4HNE can be removed or detoxified by a number of different pathways, in which the redox proteins participate lowering oxidant toxicity [15, 16].

Thioredoxin (Trx) system is present in all living cells [17] and in blood [18]. It consists of thioredoxin reductase (TrxR), Trx and NADP(H). Trx transfers electrons from TrxR to a broad range of substrates *via* its active-site Cys32 and Cys35 residues. Human cytosolic Trx (Trx1) also contains three structural Cys residues, which exhibit different responses to oxidative stress [19] and nitric oxide stress [20]. The substrates of Trx1 includes hydroperoxide [21], glutathione peroxidases that terminate lipids peroxidation cascade, and ribonucleotide reductase that is a key enzyme in DNA replication [17]. Moreover, the reduced form of Trx1 interacts with the N-terminal portion of apoptosis stimulating kinase 1 (ASK1) *in vitro* and *in vivo*, acting as a physiological inhibitor of ASK1, while the oxidation and release of Trx1 from ASK1 links cytotoxic stresses, such as TNF-α and H_2_O_2_, leading to activation of the p38 MAPK and SAPK/JNK stress response pathways [22, 23]. Thus, Trx1 system is important for cell growth/survival as well as oxidative stress-induced signal transduction [24].

Elevated Trx was found in lungs of COPD patients, which was suggested as an attempt to improve the defense against the increased oxidative stress in lung at the early stage of COPD [25]. In contrast, 4HNE production during oxidative stress was considered as a key pathway in the pathogenesis of COPD [26]. 4HNE was increased in the lungs of COPD patients [11] and in serum of AECOPD patients [27]. The cytotoxic effect of 4HNE could be resulted from its binding to the active-sites of Trx1 and cytosolic TrxR (TrxR1) [28]. So far, the pathophysiological significance of Trx1/4HNE changes in AECOPD has not been thoroughly studied. Since both Trx1 and 4HNE are also present in human serum [18, 29], the monitoring of serum Trx1 and 4HNE might be useful to assess their clinical significance that describes how alteration of serum Trx1/4HNE is related to specific properties associated with AECOPD.

Here we report a new characteristic of AECOPD: as partial pressure of oxygen (PaO_2_) drops, serum 4HNE content significantly increases and the activities of serum TrxR/Trx significantly decrease. This phenomenon is unique and not observed in patients with acute hypoxia (AH). Moreover, in the serum of AECOPD patients, the proportion of 4HNE-Trx1 adduct in total Trx1 is significantly increased. Such extracellular 4HNE and 4HNE-Trx1 adduct affect human bronchial epithelial cells *via* different mechanisms.

## Materials and methods

### Materials

Monoclonal antibodies against Trx1 or TrxR1 were purchased from Santa Cruz Biotechnology, Germany. Monoclonal antibodies against ASK-1, pASK-1, Sp-1 or Nrf-2 were purchased from Santa Cruz Biotechnology, Inc. (Santa Cruz, CA, USA). Polyclonal antibody against 4HNE was from Chemicon (for Western blotting) and Abcam (for immunoprecipitation). 4HNE was obtained from Calbiochem (USA) as a stock solution in ethanol. Insulin, DTNB, guanidine hydrochloride (Gua·HCl), phenylmethanesulfonyl fluoride (PMSF) and NADPH were purchased from Sigma-Aldrich Co. (St Louis, Mo, USA). Annexin V-FITC Apoptosis Detection Kit was purchased from Sigma-Aldrich Co. (St Louis, Mo, USA). CCK-8 Detection Kit was purchased from Dojido (Kumamoto, Japan). Reactive Oxygen Species (ROS) Assay Kit was purchased from Beyotime Biotechnology Company (Jiangsu, China). Human bronchial epithelial cell line (16HBE) was purchased from the Cell Room of Hunan in Xiangya Medical College (Hu Nan, China).

### Collection of serum samples

Serum samples were collected from 180 subjects, including 120 AECOPD patients, 38 healthy individuals and 22 patients with acute hypoxia (AH). Their clinical characteristics are present in Table 1.

**Table 1:** Clinical characteristics of study subjects.

n = 180	n	Sex (M/F)	Age (years)^a^	PaO_2_ (mmHg)^a^	PaCO_2_ (mmHg)^a^
Healthy individuals	38	27/11	66 ± 4	-	-
AECOPD (PaO_2_ > 80 mmHg)	25	16/9	71 ± 6	94.7 ± 6.9	43.66 ± 8.1
AECOPD (PaO_2_ = 60 ~ 80 mmHg)	34	26/8	69 ± 8	66.8 ± 4.7	40.13 ± 9.1
AECOPD (PaO_2_ = 40 ~< 60 mmHg)	40	27/13	68 ± 7	51.2 ± 5.5	47.03 ± 11.2
AECOPD (PaO_2_ < 40 mmHg)	21	13/8	69 ± 7	32.4 ± 1.0	64.27 ± 18.4
AH (acute hypoxia patients)	22	15/7	62 ± 8	61.7 ± 11.2	39.20 ± 7.8

^a^ Data are expressed as means ± S.D.

All participants signed the written informed consent. Shanxi Bethune Hospital Ethics Committee approved this procedure (No. 2014053). The study was performed in conformance with the Declaration of Helsinki ethical guidelines. After collection of the whole blood, the blood was allowed to clot by leaving it undisturbed at room temperature. Serum was separated as soon as possible within 1.5 h of collection. All serum samples were the remaining part after the completion of clinical laboratory tests. AECOPD patients were previously diagnosed as COPD according to GOLD criteria [forced expired volume in one second (FEV1) < 80% of predicted value and the ratio of FEV1/forced vital capacity (FVC) < 70%] [30]. AECOPD was diagnosed by sustained worsening of symptoms from the stable state, including increased breathlessness, sputum purulence or increased sputum volume [30]. Just before oxygen supplementation, serum samples were collected and an arterial blood gas analysis was performed using a blood gas analyzer (Radiometer ABL700, Denmark). Serum samples were frozen at -80°C until use. The individuals’ names and contact details had been erased from the data before they were supplied.

### Determination of serum 4HNE-protein adducts

ELISA [31] and Western blotting [32] are methods for measuring 4HNE-protein adducts. In ELISA assay, a human 4HNE ELISA kit (BG, China) was used. The assay was conducted following the protocol supplied by the manufacturer, using microplate reader (Multiskan MK3, Thermo, USA). A standard curve was used for calculating levels of 4HNE-protein adducts in the samples. In Western blotting, we carefully adjusted samples’ concentration, loaded the same amounts of total serum proteins into each lane, because no protein in serum is suitable for loading control. The positive bands were detected by chemiluminescence, and the signal intensity was analyzed using semi-quantitatively using ImageJ 1.42q.

### Cell culture

Human bronchial epithelial cell line (16HBE) was used as a model. The cells were usually cultured in minimum essential medium (MEM, Invitrogen, USA), unless specified, supplemented with 15% FBS (Xuri, China) and 100 μg/ml gentamycin sulfate (Ameresco, USA) at 37°C in an incubator containing 5% CO_2_. After 70% to 80% confluence, the cells were used for the experiments. Cell lysates were prepared through washing with PBS for 3 times, followed by harvest and ultrasonication in presence of 1 mM PMSF. The cell lysates were used freshly or stored at– 70°C until use.

### Determination of TrxR/Trx activities

TrxR/Trx activities in human serum samples and in the cell lysates were measured in 96-well plates using modified super insulin assay [18], during which an aliquot of 20 μl serum or cell lysate was used. The activity of TrxR/Trx was expressed as changes in mA_412nm_/mg protein•min. This method measures the activity of total Trx and TrxR in the cells, which include cytoplasmic Trx1/TrxR1 and mitochondrial Trx2/TrxR2.

### Western blotting

Serum samples were diluted with 1×PBS into 5 mg/ml. The cell lysates were adjusted to a protein concentration of 2 mg/ml. Serum samples (200 μg/lane) or the cell lysates (80 μg/lane) were resolved by SDS-PAGE (10% gel), then transferred onto a PVDF membrane, followed by blocking with 5% (w/v) nonfat dry milk in 50 mM Tris, pH 7.6, 150 mM NaCl and 0.1% Tween-20 (TBS-T) for 2 ~ 4 h at room temperature. Antibodies were diluted with TBS-T containing 5% nonfat dry milk. The membrane was probed with corresponding primary antibody overnight at 4°C. Antibodies of Trx1 and TrxR1 were used at 1:500 dilution, respectively. Since Trx1/TrxR1 can be secreted by cells, this study thus payed more attention to Trx1/TrxR1. Horseradish peroxidase (HRP)-linked secondary antibodies (Jackson, USA) was used at a 1:10000 dilution and incubated with membranes for 2 h at room temperature. The protein bands were visualized using enhanced chemiluminescence (ECL) system, followed by exposure to Amersham Hyperfilm MP at room temperature. The immunoblot bands were analyzed using semi-quantitatively using ImageJ 1.42q.

### Co-immunoprecipitation

First, the samples were pre-cleaned to reduce non-specific binding of proteins to IgG or agarose beads. The cell lysates (500 μg of total protein) or human serum samples (5 mg of total protein) were incubated with normal IgG and protein A/G-agarose (Beyotime, China) for 2 h, followed by centrifugation at 2500 rpm for 5 min. The resulting supernatants were incubated with 1 μg of anti-Trx1/TrxR1 antibody or 1.3 μg of anti-4HNE antibody (Abcam) overnight at 4°C, followed by addition of 20 μl protein A/G-agarose beads and incubation for another 3 h at 4°C. After centrifugation, the beads were collected, washed and boiled in SDS-PAGE sampler buffer (with or without 100 mM DTT), followed by centrifugation again. The supernatant, which contains corresponding proteins, was analyzed by Western blotting. To avoid interference from IgG bands, secondary antibody specific for IgG light chain was used for TrxR1 detection, and secondary antibody specific for IgG heavy chain was used for Trx1 detection.

### Measurement of cell viability

The cells were cultured in RPMI medium supplemented with 12% fetal bovine serum (FBS) in a CO_2_ incubator (5% CO_2_, 37°C). In brief, 8 × 10^3^ cells/well were seeded in 96 well plates and incubated for 24 h. After 70 ~ 80% confluency, the cells were exposed to the designed concentrations of 4HNE/4HNE-Trx1 adduct and incubated for 12 h at 37°C, which was followed by removing the media and washing the cells with PBS for 3 times. Then, the following two methods were used.

One is MTT assay. The pre-prepared MTT solution was added into each well till a final concentration of 500 μg/ml, and then the cells were incubated at 37°C for 4 h. Afterwards, the culture medium was carefully removed. DMSO (150 μl) was added to each well, mixed thoroughly and incubated 37°C for 10 min. The resulting absorbance at 540 nm was read using a microplate reader (Synergy-H1, BioTek, USA).

Another is cell proliferation assay (CCK-8 assay). CCK-8 kit (Dojindo, Kumamoto, Japan) was used. An aliquot (10 μl) of CCK-8 solution (10% v/v in RPMI medium) was added to each well and incubated at 37°C for 1 h. The absorbance at 450 nm was measured using a microplate reader (Synergy-H1, BioTek, USA).

### Detection of apoptosis and ROS level

The cells (2 × 10^5^ cells/well) were seeded in 6-well plates and incubated for 24h. On the following day, the cells were treated with the designed concentrations of 4HNE or 4HNE-Trx1 adduct for 12h. Subsequently, the cells were collected by centrifugation for 5 min at 2500 rpm, followed by washing with PBS for 3 times. The cells were stained with both annexin V-FITC and PI for flow cytometric analysis. The percentage of apoptotic cells was measured using a flow cytometer (Cytomics FC 500, Beckman, USA).

Alternatively, the cells were stained with 2’,7’-Dichlorodihydrofluorescein diacetate (DCFH-DA) and incubated at 37°C for 30 min in the dark. Then, the cells were washed with PBS for 3 times and applied to determine ROS level using a flow cytometer (Cytomics FC 500, Beckman, USA).

### Analyzing the mRNA levels of TrxR1 and Trx1 by real-time quantitative PCR

Total RNA was extracted from the cells using the mini-RNA Extraction Kit produced by Huashun Company of China or Trizol reagent (Invitrogen Company of America). Firstly, 1 μg RNA was used as template and reversely transcribed into cDNA using the DNA reverse transcriptase of Promega Company. TrxR1, Trx1 and β-actin specific primer pairs were designed as follows: TrxR1, (forward) 5’-GGG ACA GAA TGA TAG AAG C-3’, (reverse) 5’-CTA AAC CAA TAC CAG CAA G-3’; Trx1, (forward) 5’-GAT GTT GCT TCA GAG TGT-3’, (reverse) 5’-AAC TGG GTT TAT GTC TTC-3’; β-actin, (forward) 5’-CTC CAT CCT GGC CTC GCT GT-3’, (reverse) 5’-GCT GTC ACC TTC ACC GTT CC-3. The DNA was mixed with SYBR green Real-Time PCR Master Mix produced by Toyobo Company in Japan to carry out RT-PCR. The specific operation was as follows: pre-denaturation at 95°C for 10 min, 95°C for 30 sec, 55°C for 1 min, 72°C for 30 sec, 40 cycles (Bio-Rad-Cycler RT-PCR analyzer, USA). The relative expression of TrxR1 and Trx1 genes was calculated by software *via* comparing the expression of TrxR1 and Trx1 genes with that of internal reference gene (β-actin).

### Proteomics analysis

The peptide samples were prepared by filter assisted sample preparation (FASP) method. Briefly, protein samples were incubated with dithiothreitol (DTT) to a final concentration of 5 mM for 30 min at 56°C, then iodoacetamide (IAA) was added to a 20 mM final concentration, which was incubated in the dark at room temperature. After 0.5 h, the samples were mixed with 5 mM final concentration of DTT and kept in the dark for another 15 min. Afterwards, protein samples were loaded into 10 kDa Microcon filtration devices (Millipore) and centrifuged at 12,000 g for 20 min and washed twice with urea lysis buffer (8 M Urea, 100 mM Tris-HCl pH 8.0), twice with 50 mM NH_4_HCO_3_. Then the samples were digested using trypsin at an enzyme to protein mass ratio of 1:25 overnight at 37°C. The resulting peptides were extracted, and dried down (SpeedVac, Eppendorf).

Orbitrap Fusion LC-MS/MS analyses were performed on an Easy-nLC 1000 liquid chromatography system (Thermo Fisher Scientific) coupled to an Orbitrap Fusion *via* a nano-electrospray ion source (Thermo Fisher Scientific). The tryptic peptides were dissolved with loading buffer (acetonitrile and 0.1% formic acid). The tryptic peptides were eluted from a 150-μm ID × 2 cm, C18 trap column and separated on a homemade 150 um ID × 12 cm column (C18, 1.9 μm, 120 Å, Dr. Maisch GmbH) with a series of adjusted linear gradients according to the hydrophobicity of fractions at a flow rate of 500 nl/min. Survey scans were acquired after the accumulation of 5e5 ions in the Orbitrap for m/z 300—1,400 using a resolution of 120,000 at m/z 200. The top speed data dependent mode was selected for fragmentation in the HCD cell at normalized collision energy of 32%, and then fragment ions were transferred into the ion trap analyzer with the AGC target at 5e3, maximum injection time at 35 ms. The dynamic exclusion of previously acquired precursor ions was enabled at 18 s.

Spectral data were searched against human protein RefSeq database (2013.07.01) in Proteome Discoverer1.4.1.14 suites with Mascot software (version 2.3.01, Matrix Science) to achieve a false discovery rate of < 1%. The mass tolerance was set to be 10 ppm for precursor, and it was set 0.5 Da for the tolerance of product ions. Two missed cleavage sites for trypsin was allowed.

Bioinformatics Analysis Gene Ontology annotation was performed using the online tool PANTHER software 13.1 (http://www.panther.org) and DAVID software 6.8 (http://www.David.gov.).

### Statistical analysis

Statistical data were expressed as means ± standard deviation (S.D.). In serum experiments, One-way ANOVA was used to compare the mean values of different groups. Since we obtained a significant value of omnibus F-test, a Dunnett’s post-hoc test was then used to determine which subgroup’s values were significantly different from control subjects. Correlations among serum 4HNE concentration, serum TrxR/Trx activities, PaO_2_ and PaCO_2_ were evaluated using Spearman’s correlation test. Statistical analyses were performed using SPSS software, version 18.0. *P*-value of less than 0.05 was considered as statistical significance, and *P*-value of less than 0.01 was considered as extremely significance.

## Results

### Decreased serum TrxR/Trx plus increased serum 4HNE was unique for AECOPD

As shown in Table 1, 120 serum samples were collected from AECOPD patients, which were classified into four groups based on partial pressure of O_2_ (PaO_2_). These AECOPD patients had been excluded from pneumonia, asthma, lung cancer, interstitial lung disease and cardiovascular disease. Control serum samples were collected from 38 healthy individuals who had annual health check in the Hospital Health Medical Center. 22 serum samples were collected from the patients with acute hypoxia (AH). These AH patients had normal lung function but suffered from acute abdominal disease, acute myocardial infarction or acute brain disease.

**Fig 1:**
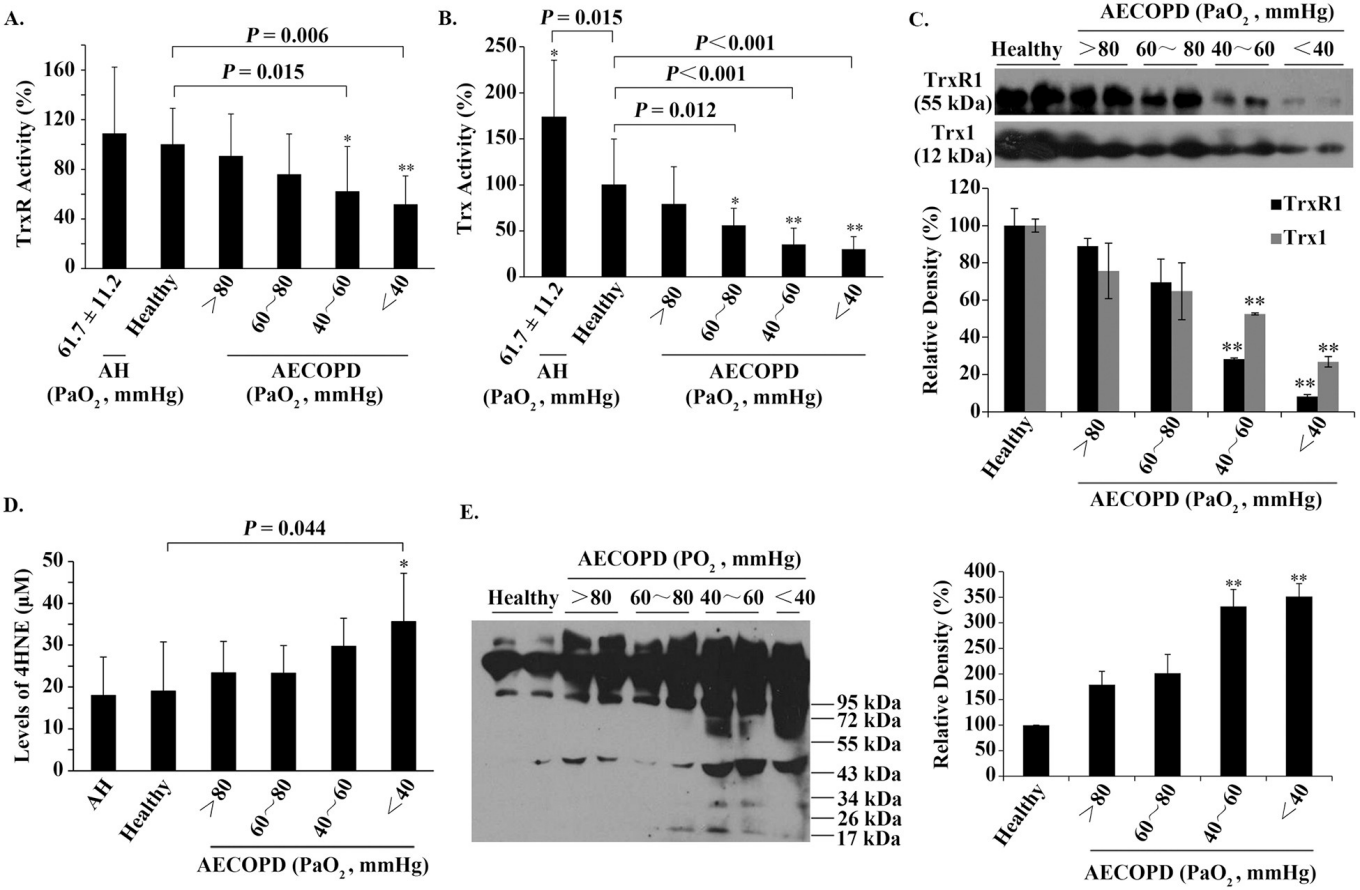
The relationships among serum TrxR/Trx, serum 4HNE and PaO_2_. (**A**) The relationship between the activity of serum TrxR and PaO_2_. (**B**) The relationship between the activity of serum Trx and PaO_2_. (**C**) Representative Western blots (upper panel) and relative density values (bottom panel) for the bands in the upper panel. (**D**) The relationship between the level of serum 4HNE and PaO_2_. (**E**) Representative Western blots (left panel) and relative density values (right panel) for the bands in the left panel. In the bar graphs, the values are expressed as mean ± S.D. (n ≥ 3). *, *P* < 0.05; **, *P* < 0.01 *versus* healthy control.

No significant differences were observed in their age distribution (*P* = 0.798). Given that increased hypoxemia is closely related with COPD disease severity [33], we categorized 120 AECOPD patients according to their PaO_2_ values (Table 1). Compared to healthy people, AECOPD patients had a significant decrease in serum TrxR activity (*P* < 0.05) as PaO_2_ decreased to < 60 mmHg and a significant decrease in serum Trx activity (*P* < 0.05) upon PaO_2_ decreased to < 80 mmHg, whereas AH patients had no significant change in serum TrxR activity but had significant enhancement in serum Trx activity (*P* = 0.015), as shown in Fig 1A and 1B.

These data suggest that only hypoxia caused by AECOPD can lead to a decrease in the activities of serum TrxR/Trx. Western blotting revealed that the protein levels of serum TrxR1/Trx1 were also progressively decreased in parallel with the reduction of PaO_2_ in AECOPD patients (Fig 1C). Both the activities and proteins of serum TrxR/Trx showed a significant decrease when PaO_2_ was below 60 mmHg (*P* < 0.05) (Fig 1A–1C).

In contrast, the levels of serum 4HNE-protein adducts, measured by ELISA, were 18.04 ± 9.14 μM for AH patients and 19.06 ± 11.73 μM for healthy people. The difference between them did not reach a statistical significance (Fig 1D). However, as PaO_2_ decreased from > 80 mmHg to < 40 mmHg, the values of serum 4HNE-protein adducts increased from 23.37 ± 7.53 μM to 35.63 ± 11.56 μM for AECOPD patients, in which a significant increase was observed at PaO_2_ below 40 mmHg (Fig 1D). These values of serum 4HNE are close to those of plasma 4HNE [34].

To see the molecular weight of serum 4HNE-protein adducts, equal amounts of serum samples (150 mg total protein) were separated by non-reducing SDS-PAGE, followed by Western blotting using an anti-4HNE polyclonal antibody. In the absence of DTT, the prominent 4HNE-positive species had molecular weight over 55 kDa, the amounts of which were increased gradually as PaO_2_ reduced (Fig 1E), which was basically consistent with the result obtained from ELISA (Fig 1D).

### The fall of PaO_2_ in AECOPD as a potential cause for elevating serum 4HNE/reducing serum Trx

The potential correlation among serum TrxR/Trx activities, serum 4HNE level, PaO_2_ and PaCO_2_ in AECOPD patients were analyzed by Spearman’s Rank Correlation Test. PaO_2_ was positively correlated with the activities of serum TrxR/Trx, and negatively correlated with serum 4HNE level, among which its correlation with TrxR activity was not statistically significant (*P* = 0.45), and its correlation with Trx activity had statistical significance (*P* = 0.015), indicating that serum TrxR is not as sensitive to the changes in PaO_2_ as serum Trx. PaCO_2_ was negatively correlated with the activities of serum TrxR/Trx and PaO_2_, but positively correlated with serum 4HNE level. Among them, only the correlation of PaCO_2_ with PaO_2_ was statistically significant (*P* = 0.003), suggesting that PaCO_2_ has no direct relationship with changes in serum TrxR/Trx and 4HNE. Serum 4HNE level was negatively correlated with PaO_2_ value (*P* = 0.013) and negatively correlated with activities of serum TrxR/Trx, in which its correlation with Trx activity was statistically significant (*P* = 0.021) (Table 2).

**Table 2:** Correlations of serum 4HNE, serum TrxR/Trx activities, PaO_2_ and PaCO_2_ in AECOPD patients.

	TrxR activity			Trx activity			4HNE			PaO_2_		
	*r*^a^	P	n^b^	*r*^a^	P	n^b^	*r*^a^	P	n^b^	*r*^a^	P	n^b^
PaO_2_	0.102	0.450	57	**0.326**	**0.015**	55	**- 0.521**	**0.013**	22		-	
PaCO_2_	- 0.015	0.921	47	- 0.201	0.141	55	0.281	0.231	20	**- 0.324**	**0.003**	80
4HNE	- 0.141	0.456	30	**- 0.590**	**0.021**	15		-		**- 0.521**	**0.013**	22

^a^ Spearman’s correlation coefficient.

^b^ Number of samples.

Bold font indicates statistical significance.

These results prove that serum Trx is more closely related to 4HNE than serum TrxR. Overall, the activity of serum TrxR was not sensitive to changes in PaO_2_, PaCO_2_ and serum 4HNE level; the activity of serum Trx significantly increased as PaO_2_ elevated but decreased when 4HNE level enhanced; the negative correlation between 4HNE and PaO_2_ was statistically significant, but the positive correlation of 4HNE with PaCO_2_ was not statistically significant. Therefore, a main cause for an enhancement in serum 4HNE level and a decrease in serum Trx activity seems to be the fall of PaO_2_, while PaCO_2_ has no direct relationship with changes in serum TrxR/Trx and 4HNE.

### Aggregation of serum 4HNE-positive species formed mainly *via* disulfide bonds

**Fig 2:**
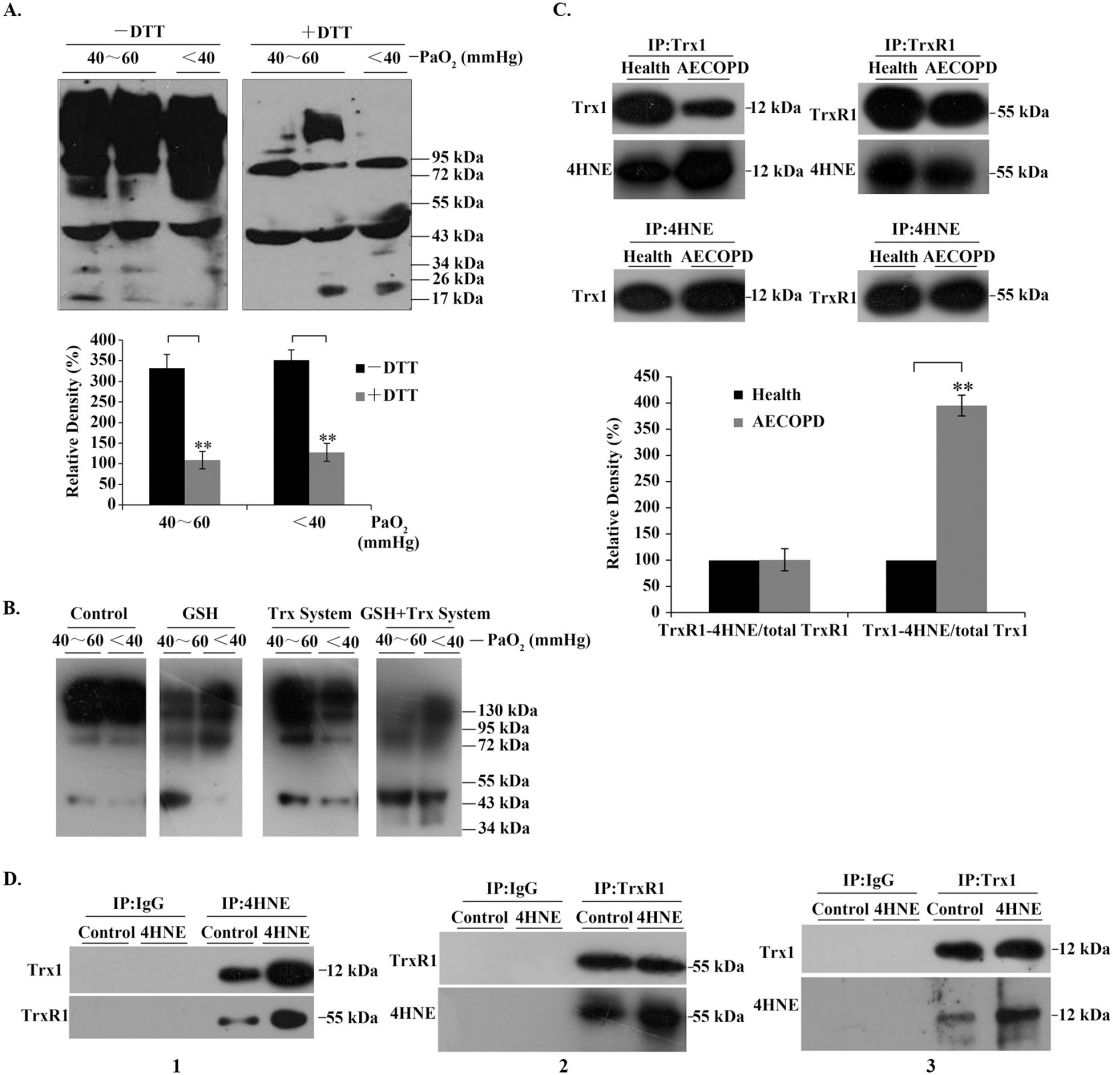
4HNE-protein adducts in serum and in bronchial epithelial cells. (**A**) 4HNE-protein adducts in human serum. Representative Western blots (upper panel) and relative density values (bottom panel) for the bands in the upper panel. (**B**) Representative Western blots. Control, buffer; GSH, 1mM; Trx system (5 μM Trx1, 100 nM TrxR1 and 0.2 mM NADPH), GSH + Trx system (1 mM GSH plus 5 μM Trx1, 100 nM TrxR1 and 0.2 mM NADPH). The serum samples were incubated with the above agents, respectively, for 90 min at room temperature. (**C**) Representative Western blots (upper panel) and relative density values (bottom panel) for the bands in the upper panel. 4HNE-TrxR1/Trx1 adducts were detected by Western blotting after co-immunoprecipitation of them from human serum samples. (**D**) Representative Western blots. The cells were treated with 50 μM 4HNE for 6 h, and the 4HNE-TrxR1/Trx1 adducts in the cells were detected by Western blotting after immunoprecipitation (IP). In the bar graphs, the values are expressed as mean ± S.D. (n ≥ 3). **, *P* < 0.01.

As the formation of HNE-protein adducts is one of the accompanying processes in oxidative stress or redox imbalance [35], we examined the effect of reducing agents on 4HNE-protein adducts. After incubation of the serum samples with 100 mM DTT for 30 min, most of 4HNE-positive species with high-molecular-weight disappeared (Fig 2A).

Through analyzing the relative density of the positive bands, we found that about 75% of serum 4HNE-positive species formed intermolecular disulfide bonds in AECOPD patients with PaO_2_ below 60 mmHg (lower panel in Fig 2A). Similarly, antioxidant molecules present in the body, such as 1 mM GSH or Trx1 system, could partially resolve the high-molecular-weight species, which was more efficient when Trx1 system was used in combination with GSH (Fig 2B). All these molecules can open disulfide bonds.

### The presence of 4HNE-TrxR1/Trx1 adducts in serum/human bronchial epithelial cells

Using co-immunoprecipitation (Co-IP) and Western blot analysis, we found that the serum 4HNE-protein adducts contained 4HNE-TrxR1/Trx1 adducts (upper panels, Fig 2C), in which the proportion of 4HNE-Trx1 adduct in the total Trx1 was significantly increased, while the amount of 4HNE-TrxR1 adduct did not change in AECOPD patients, as compared with those in healthy people (lower panels, Fig 2C). Interestingly, in human bronchial epithelial cells, the addition of extracellular 4HNE led to an increase not only in cellular 4HNE-Trx1 adduct but also 4HNE-TrxR1 adduct (panel 1, Fig 2D); an increase in the proportion of 4HNE-TrxR1 adduct/total TrxR1 (panel 2, Fig 2D) and an increase in the proportion of 4HNE-Trx1 adduct/total Trx1 (panel 3, Fig 2D). These data indicate that unlike serum TrxR1, intracellular TrxR1 is as easily modified by 4HNE as Trx1. Since serum 4HNE may be present either as free form or as Trx1-bound form, which were increased significantly during AECOPD, and airway epithelial cells play a key role in the pathogenesis of AECOPD, we next examined the effects of 4HNE and 4HNE-Trx1 adducts on human bronchial epithelial cells, respectively.

### Effect of extracellular 4HNE on human bronchial epithelial cells

**Fig 3:**
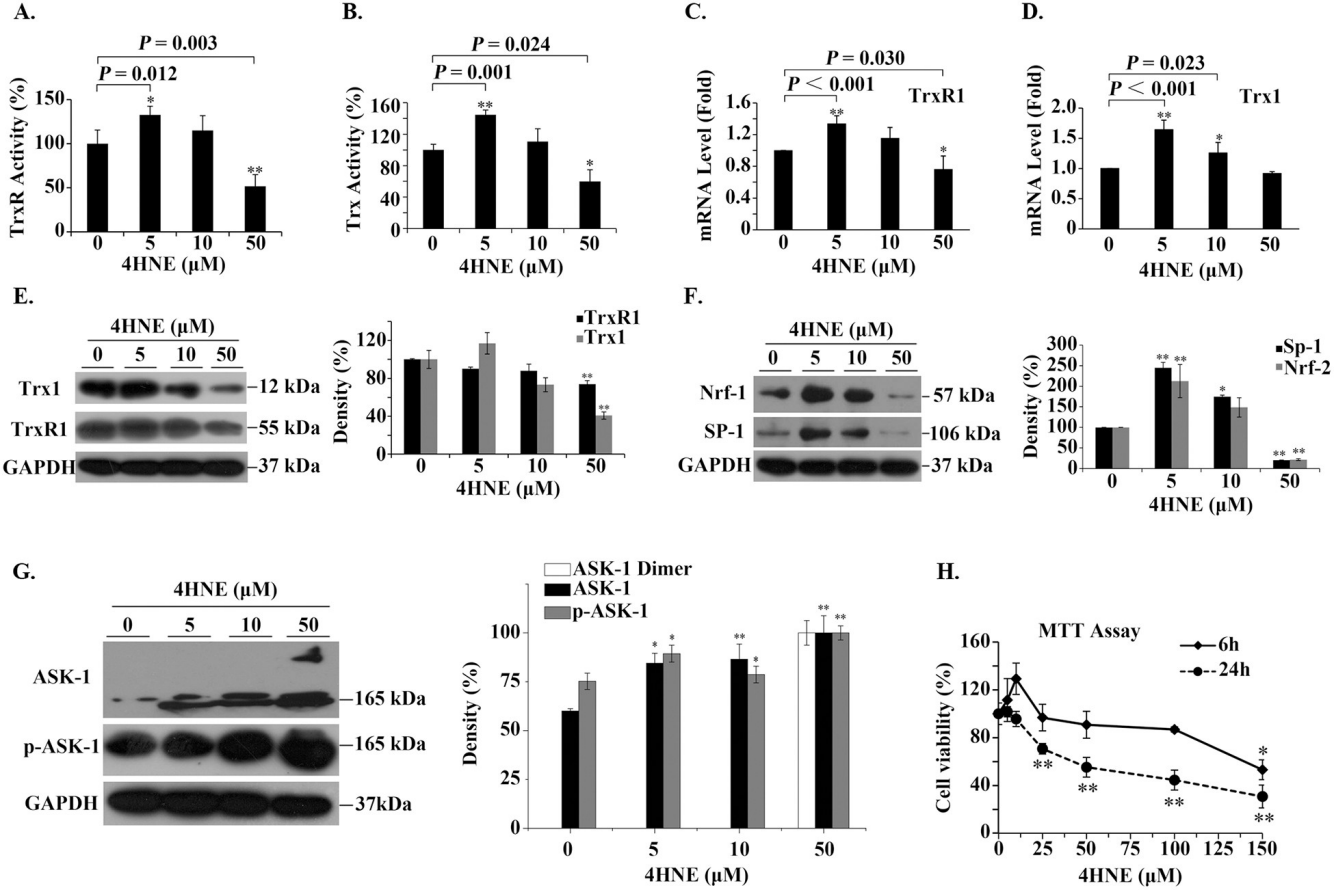
The effect of extracellular 4HNE on human bronchial epithelial cells. (**A,B**) The effect of extracellular 4HNE on the activities of cellular TrxR/Trx. The method used measures the total activity of TrxR/Trx. (**C,D**) The effect of extracellular 4-HNE on the transcription of TrxR1/Trx1. (**E,F,G**) Representative Western blots (left panel) and relative density values (right panel) for the bands in the left panel. GAPDH was used as an internal reference. In the bar graphs, the values are expressed as mean ± S.D. (n ≥ 3). (**H**) Cell viability was affected by 4HNE concentration and incubation time. *, *P* < 0.05; **, *P* < 0.01 *versus* control without 4HNE treatment.

After the cells treated with 4HNE for 6 h, 5 μM 4HNE significantly increased the activities of cellular TrxR/Trx, but 50 μM 4HNE significantly decreased the activities of cellular TrxR/Trx (Fig 3A and 3B); the mRNA levels of TrxR1/Trx1 also showed the similar changes that increased at 5 μM 4HNE and decreased at 50 μM 4HNE (Fig 3C and 3D); in the presence of 5 μM 4HNE, Trx1/TrxR1 protein contents did not significantly increase, but they both significantly decreased in the presence of 50 μM 4HNE (Fig 3E).

Since the transcription factor nuclear factor erythroid-2 related factor 2 (Nrf-2) may affect Trx1 transcription [36] and the transcription factor Sp-1 can enhance Trx1 transcription [37], we examined the effect of 4HNE on them. Their response was biphasic. Their expression significantly increased with 5 μM 4HNE, then decreased with increasing 4HNE concentration (Fig 3F). These data together suggest that 4HNE also affect transcription of TrxR1/Trx1 besides 4HNE inhibits TrxR1/Trx1 activities through binding to their active-site residues [28].

Since Trx1 inhibits apoptosis signal-regulating kinase 1 (ASK1)-nduced apoptosis [38], we test whether 4HNE is involved in the activation of ASK1. After the cells treated with 5 ~ 50 μM 4HNE for 6 h, the levels of ASK1 and phosphorylated ASK1 (pASK1) were significantly increased. Especially, in the cells treated with 50 μM 4HNE (Fig 3G), ASK1 dimer appeared. These results are consistent with that ASK1 activation is regulated by multiple steps including self-dimerization, phosphorylation, and protein-protein interactions. This suggests that when high concentrations of 4HNE cause a decrease in Trx1 content, the unbound form of ASK1 increases. ASK1 may be subsequently activated by oligomerization and the phosphorylation of a critical threonine residue [39].

**Fig 4:**
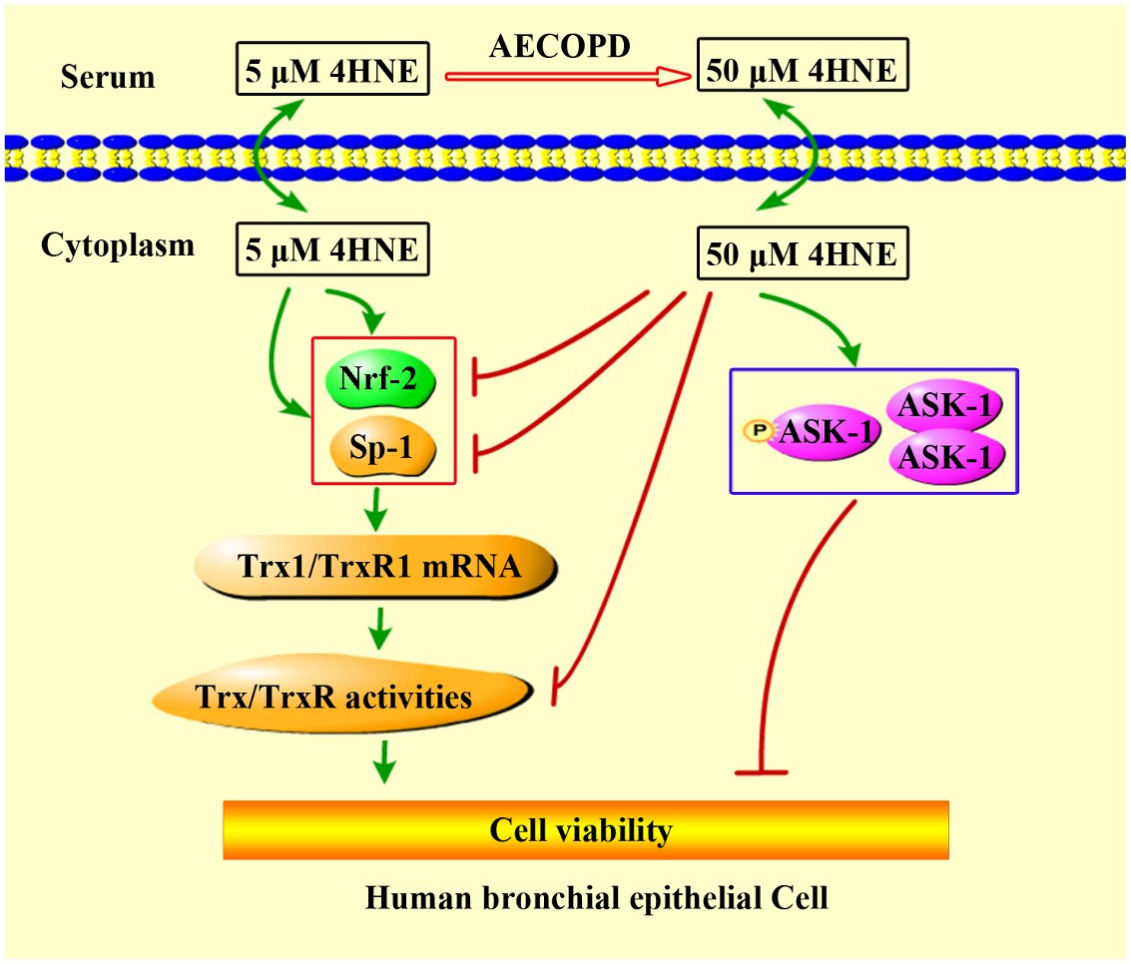
Proposed mechanism for extracellular 4HNE to affect cell viability. 4-HNE can pass through cell membrane freely. The low concentration of extracellular 4HNE promotes cell viability, which was consistent with its ability to promote TrxR1/Trx1 expression and activities. TrxR1 and Trx1 are critical to cell survival [40]. High concentrations of extracellular 4HNE, such as in the case of AECOPD patients, show cytotoxicity, which is, at least in part, related to the inhibition of 4HNE on TrxR/Trx as well as the activation of ASK1.

To investigate the possibility that the changes in cell viability were related to the changes in cellular Trx1 content, MTT assay was conducted. We found that upon treatment with 4HNE for 6 h, the cell viability showed a three-stage response: it increased as concentration of 4HNE increased till < 25 μM, then it remained stable when concentration of 4HNE was 25 ~ < 100 μM, if 4HNE concentration was greater than 100 μM, the cell viability began to decrease (Fig 3H). However, when the incubation time of 4HNE with cells was extended to 24 h, the cell viability remained unchanged or even slightly increased only when 4HNE concentration was less than 10 μM, and then decreased linearly with the increase of 4HNE concentration (Fig 3H). The longer 4HNE was incubated with cells, the more sensitive the cells were to 4HNE. However, low concentration of 4HNE not only promotes Trx activity but also promotes cell viability. For clarity, these results are outlined in Fig 4.

### Cellular Trx1 conferred the cells resistance to low concentrations of 4HNE

**Fig 5:**
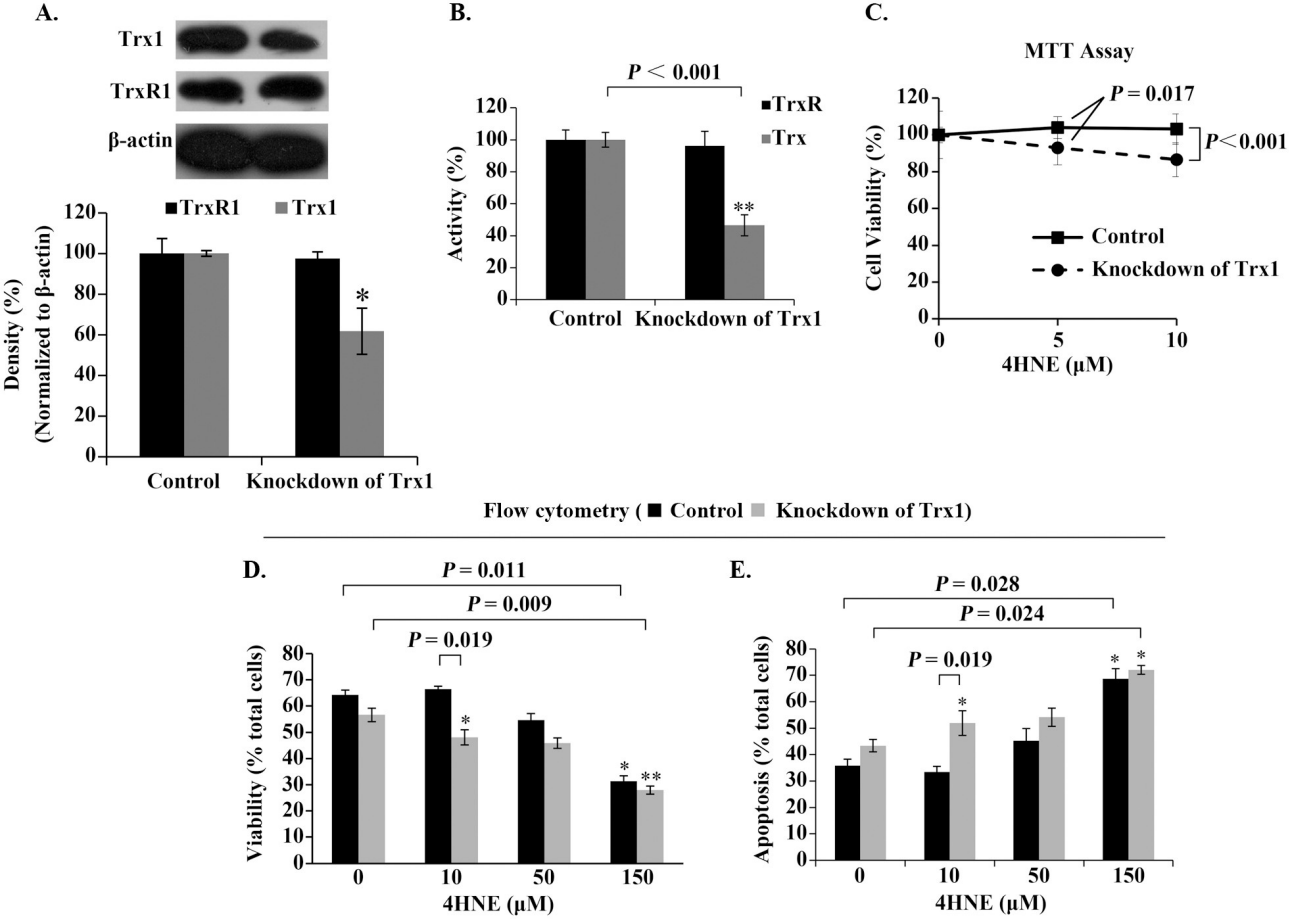
Role of cellular Trx1 in mediating the effect of extracellular 4HNE on cell survival. (**A**) Analysis of Trx1-knockdown efficiency. β-actin was used as an internal reference. Representative Western blots (upper panel) and relative density values (bottom panel) for the bands in the upper panel. (**B**) Activities of cellular Trx/TrxR. (**C**) Trx1-Knockdown decreased the ability of the cells against 4HNE. (**D,E**) Analyses of the cell viability and death by flow cytometer. In the bar or curve graphs, the values are expressed as mean ± S.D. (n ≥ 3). *, *P* < 0.05; **, *P* < 0.01.

Following this reasoning, we would like check whether cellular Trx1 will critically influence the effect of 4HNE on cell viability. The cellular Trx1 was efficiently knocked down by short hairpin RNA (shRNA) (Fig 5A and 5B).

MTT test indicated that the cells with Trx1-deficiency were no longer resistant to low concentrations of 4HNE (Fig 5C). The results from flow cytometry also showed that 10 μM 4HNE inhibited rather than promoted the viability of the Trx1-deficient cells (Fig 5D and 5E). These results confirm that cellular Trx1 contributes cell resistance to low concentrations of 4HNE.

### Effect of extracellular 4HNE-Trx1 adduct on human bronchial epithelial cells

To detect the effect of extracellular 4HNE-Trx1 adducts on the cells at the proteomic level and to explore more information, label-free quantitative proteomics analysis was carried out. Since serum Trx1 is at nanomolar concentrations ranging from 6.79 ng/ml (0.566 nM) to 132.9 ng/ml (11.08 nM) [18, 41–47], the cells were treated with 100 nM 4HNE-Trx1 adduct or Trx1 for 12 h. The cell samples from 3 cultures were combined for proteomic analysis. A total of 2332 proteins were detected in the samples treated with 4HNE-Trx1 adduct plus the samples treated with Trx1. Of them, the threshold of ≥ 2 Unique Peptides [48] and the cutoff threshold ≥ 5-fold change were used to define the differentially expressed proteins, 92 proteins were finally selected for further analysis (S1 Table).

**Fig 6:**
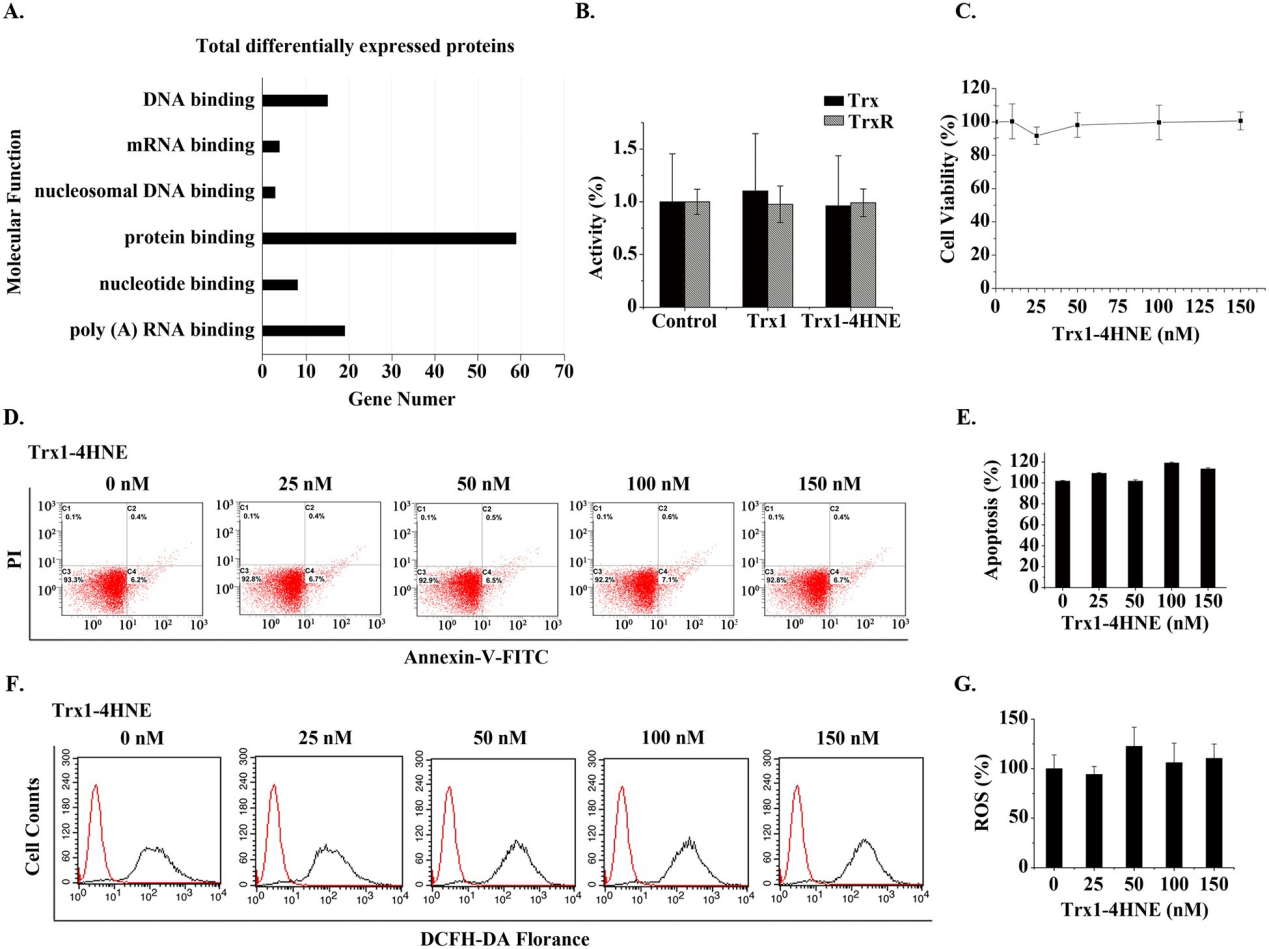
Effect of extracellular 4HNE-Trx1 adduct on the cells. (**A**) Gene ontology analysis of ≥ 5-fold differentially expressed proteins based on their molecular functions using DAVID software. (**B**) Activities of cellular Trx and TrxR. The data represent mean ± S.D. of results from three independent experiments. (**C**) CCK-8 assay. The effect of extracellular 4HNE-Trx1 adduct on the cell viability. (**D,E**) Apoptotic assay. Result of flow cytometry (left panel) and flow cytometry histograms (right panel). The result was a representative of three independent experiments. (**F,G**) Assay of ROS levels. Result of flow cytometry (left panel) and flow cytometry histograms (right panel). The result was a representative of three independent experiments. In the bar graphs, the values are expressed as mean ± S.D. (n ≥ 3).

Of this 92 differentially expressed proteins, 48 proteins were up-regulated and 44 proteins were down-regulated in the samples treated with 4HNE-Trx1 adduct *vs* the samples treated with Trx1. Using DAVID classification, the 92 proteins were categorized by their biological processes, molecular function and cellular component. In general, the differentially expressed proteins were binding proteins (Fig 6A), among which the predominant proteins were involved in RNA metabolism, including transcription regulation, pre-mRNA splicing process, mature mRNA stability, translation, mRNA decay, and ribosome biogenesis (S1 Table).

For instance, GTF2H1, GTF3C1 and GTF3C5 are related to transcription regulation; DDX23, DDX41, CELF1, ZNF326 and PPP1R8 are involved in mRNA splicing; UPF3B is related to the nonsense mediated decay (NMD) of mRNA.

In addition, 4HNE-Trx1 adduct induced an increase in the expression of NDUFS7 and OXA1L. NDUFA7 [mitochondrial NADH dehydrogenase (ubiquinone) iron-sulfur protein 7] is the core subunit of NADH dehydrogenase (complex I) in mitochondrial respiratory chain. Complex-I plays a role in electron transfer from NADH to the respiratory chain. OXA1L (mitochondrial inner membrane protein) is essential for the activity and assembly of cytochrome oxidase, which is necessary for the correct biosynthesis of mitochondrial ATPase and Complex-I. The increase in these two proteins is expected to enhance the electronic transfer *via* respiratory chain. However, the proteomic results showed that there were no significant changes in other redox proteins and the proteins involved in apoptosis. Consistently, such a treatment did not cause a significant change in the activities of cellular TrxR and Trx (Fig 6B), in the cell viability (Fig 6B), in apoptosis (Fig 6D and 6E) and in ROS level (Fig 6F and 6G). These data indicate that the mechanism for extracellular 4HNE-Trx1 adducts to affect the cells is different from that of extracellular 4HNE.

## Discussion

The results of this study have revealed that the decrease in PaO_2_ caused by AECOPD contributes to significant decrease in the activities of serum TrxR/Trx and increase in the level of serum 4HNE, whereas the decrease in PaO_2_ caused by AH has little effect on serum TrxR activity and serum 4HNE level, but significantly promotes serum Trx activity. These results suggest that the decreased PaO_2_ is not a single factor that causes a decrease in the activities of serum Trx/TrxR during AECOPD. It seems likely that decreased PaO_2_ causes 4HNE to increase, the increased 4HNE in turn leads to decreasing Trx. Accordingly, the elevation of 4HNE in the serum of AECOPD patients (Fig 1D and 1E) is supposed to be a key step in decreasing Trx1. This assumption can be supported by the observation that an increase in serum Trx during acute hypoxia is in parallel with normal level of serum 4HNE (comparing Fig 1B with Fig 1D).

In AECOPD, serum 4HNE level is significantly and negatively related to PaO_2_ value, but is proportional to PaCO_2_. The latter is not statistically significant. This result suggests that high level of serum 4HNE in AECOPD patients is mainly related to decreased PaO_2_ rather than increased PaCO_2_. Moreover, serum Trx activity rather than TrxR activity is significantly and positively related to PaO_2_ values, but significantly and negatively associated with serum 4HNE levels, indicating that a decrease in serum Trx activity plus an increase in serum 4HNE is a potential marker for reflecting the severity of AECOPD. However, many functions of TrxR1 are dependent on Trx1 [49]. Thus, the remarkable decline in Trx1 will undoubtedly impair functions of Trx1 system.

Protein aggregation plays a potential role in the pathology of COPD [50]. We have found the elevated aggregation of 4HNE-protein adducts in the sera of AECOPD patients. These 4HNE-positive aggregates with high-molecular-weight significantly dissolved upon incubation with 10 mM DTT or 1 mM GSH plus Trx1 system, demonstrating that the 4HNE-related protein aggregates are held together mainly *via* disulfide bonds, and the reducing molecules can prevent 4HNE-protein adducts from aggregation. Thus, the decrease of serum TrxR1/Trx1 in AECOPD patients may contribute, at least partially, to 4HNE-associated protein aggregation because Trx1 system has the ability to reduce disulfide [49] and converse GSSG into GSH [51].

In the serum of AECOPD patients, the proportion of 4HNE-Trx1 adduct accounted for the total Trx1 content was significantly increased, while the proportion of 4HNE-TrxR1 adduct accounted for the total TrxR1 content did not change. This phenomenon should be related to the easy binding of 4HNE with thiol/selenol groups [28]. In the extracellular space with oxidizing environment, serum Trx1 and TrxR1 are usually present in the non-reduced forms [18]. Under this condition, the non-reduced form of Trx1 has exposed thiol group (Cys73) [52], while the non-reduced form of TrxR1 has no thiol/selenol groups on its surface (Zhong, unpublished result). Therefore, the concentration of serum 4HNE-Trx1 adduct, but not serum 4HNE-TrxR1 adduct, significantly enhances with the increase of 4HNE level. In a reducing environment within the cells, Trx1/TrxR1 may exit in the reduced forms that contain the exposed thiol/selenol groups. Thus, the contents of both 4HNE-Trx1 adduct and 4HNE-TrxR1 adduct increase with the elevation of 4HNE concentration. Moreover, in the 4HNE-treated cells, the content of Trx1 or TrxR1 tended to decrease, which fits well with the observation that high levels of serum 4HNE-protein adducts are companied by low levels of serum TrxR1/Trx1.

Serum TrxR1 and Trx1 are secreted by cells [53, 54]. Thus, the low levels of serum TrxR1/Trx1 in AECOPD patients with a PaO_2_ of ≤ 60 is most likely due to the decreased synthesis of Trx1 and TrxR1 in cells. Especially, serum level of 4HNE is dynamically increased from 23.37 ± 7.53 μM to 35.63 ± 11.56 μM with the decreased PaO_2_ values in AECOPD. These concentrations of 4HNE are high enough to induce the toxic effects *in vivo*, such as cell growth inhibition and apoptosis [55]. 4HNE can inhibit the activity of cellular TrxR1/Trx1 through binding to their active sites [28]. Moreover, high concentration of 4HNE exhibits the negative effects on transcription factors Nrf-2/Sp-1, on mRNA synthesis of TrxR1/Trx1 and on protein levels of TrxR1/Trx1. In short, 4HNE not only inhibits TrxR1/Trx1 activities but also inhibits their synthesis. These molecular mechanisms at least partially explain the fact that the increase in serum 4HNE is accompanied by the decrease in TrxR/Trx levels. Since Trx1 is essential for DNA replication and cell proliferation *via* transferring electrons to ribonucleotide reductase [49], and TrxR1 is the well-known enzyme that catalyzes the reduction of Trx1 by NADPH, thus 4HNE-induced changes in TrxR1 and Trx1 have important mechanistic implication for cell viability.

We have reasoned that Trx1 inhibits ASK1-induced apoptosis [38], thus 4HNE-induced decrease in Trx1 might promote ASK1 activation. Indeed, 4HNE induced an increase in the levels of ASK1, phosphorylated ASK1 and ASK1 dimer in a dose-dependent manner. The phosphorylation and dimerization of ASK1 are related to the activation of apoptosis signal [56]. This finding is consistent with that high levels of 4HNE produced time- or dose-dependent killing of the cells. Although the cell viability was completely protected if 4HNE concentration was less than 25 μM with 6 h treatment or 4HNE concentration was less than 5 μM with 24 h treatment (Fig 3H), the efficacy of low 4HNE concentration-induced cell death was enhanced upon the knock-down of cellular Trx1. This result suggests that the effect of 4HNE on cell viability is, at least partially, dependent on cellular Trx1. In addition, under the condition with 5 μM 4HNE, the protein levels of TrxR1 and Trx1 did not significantly increase like their mRNA levels. This observation can be extended to increased degradation of 4HNE-modified TrxR1/Trx1, because the modification of protein by 4HNE may initiate protein degradation *via* the ubiquitin-proteasome system [57].

Moreover, serum 4HNE-Trx1 adduct significantly increased in the case of AECOPD. Unlike extracellular 4HNE, extracellular 4HNE-Trx1 adduct does not affect the activities of cellular Trx/TrxR, cell viability, cell apoptosis and cellular ROS levels, but causes changes in the proteome of the cells. These results suggest that the effect of extracellular 4HNE-Trx1 adduct on the cells is different from that of free 4HNE.

The largest number of differentially expressed proteins is related to RNA metabolism, including nonsense-mediated decay (NMD) of mRNAs and mRNA splicing. NMD of mRNAs is a quality control pathway that eliminates mRNAs containing premature translation-termination codons (PTCs) [58]. Some inherited human diseases are due to the presence of PTC or frameshifts that induce nonsense codons in mRNAs, such as human UPF3B mutation may cause mental diseases, affect neural development and cause tumors [59–61]. UPF3B is a component of NMD pathway and is down regulated in the cells treated with 4HNE-Trx1 adduct. Under this condition, impaired cell functions cannot be ruled out.

mRNA Splicing is a major regulatory component in higher eukaryotes. Disorders in mRNA splicing are a major contributor to human disease. In fact, approximately one in three hereditary disease alleles are believed to cause aberrant splicing [62]. Therefore, down-regulated expression of the proteins related to RNA splicing in the cells treated with 4HNE-Trx1 adduct has important mechanistic implication. Therefore, it is possible that long-term elevation of extracellular 4HNE and 4HNE-Trx1 adduct in AECOPD patients may damage airway tissues and cells.

**Fig 7:**
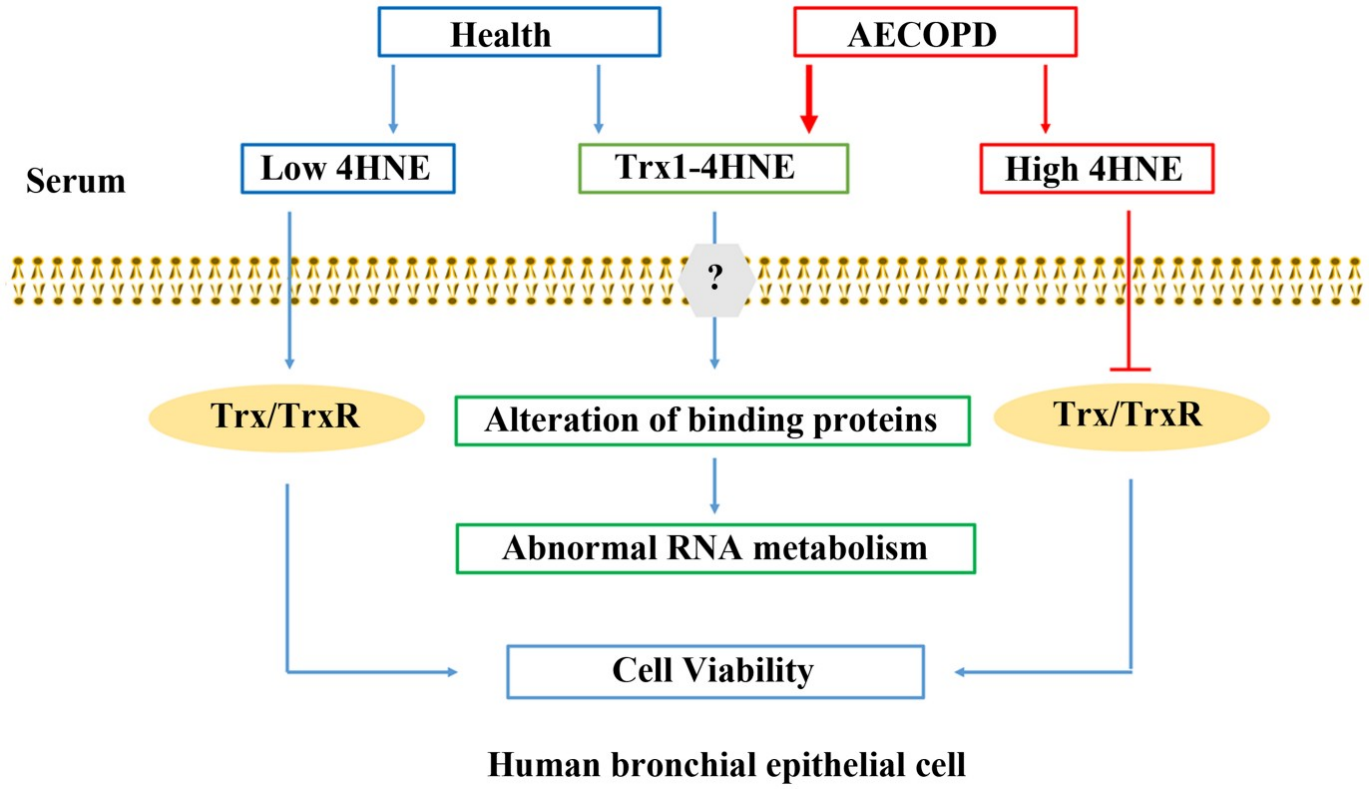
Summary of 4HNE and 4HNE-Trx1 adduct affecting human bronchial epithelial cells. In human serum, 4HNE exists in both free form or bound form. Under the condition with AECOPD, the contents of 4HNE and 4HNE-Trx1 adduct significantly increase. Low levels of extracellular 4HNE facilitates cell viability *via* elevation of cellular TrxR1/Trx1, whereas high levels of 4HNE are harmful to the cells *via* inhibition of cellular TrxR1/Trx1. 4HNE-Trx1 adduct does not decrease cell viability, and yet interferes with RNA metabolism. Question mark means that the mechanism by which cell membrane molecules react with 4HNE-Trx1 adduct is still unclear.

One explanation of the discrepancy between the action of 4HNE and the action of 4HNE-Trx1 adduct on the cells may be due to that they enter the cell by different mechanisms. 4HNE is highly diffusible, while Trx1 seems to need the help of a receptor to pass through the cell membrane [46] (Fig 7). Although the precise mechanism by which extracellular 4HNE-Trx1 adduct enters bronchia epithelial cells is largely unknown, the differentially expressed proteins are likely to associate with the airway disorder in AECOPD, which needs further study.

Since the functions of serum/cellular TrxR1/Trx1 are limited by high concentration of serum 4HNE, it is reasonable that amelioration of high 4HNE-mediated cytotoxicity by reducing oxidative stress has therapeutic implications [63].

## Conclusions

The elevation of serum 4HNE plus the decrease in the activities of serum TrxR1/Trx1 appears to be a unique characteristic of AECOPD, in which Trx1 has a closer relationship with 4HNE than TrxR1. This finding supports the notion that oxidative stress is closely related to the occurrence and development of AECOPD [64]. Moreover, serum 4HNE-Trx1 adduct is significantly increased in AECOPD patients. Both extracellular 4HNE and 4HNE-Trx1 adducts can affect human bronchial epithelial cells, but their mechanism of action is different. It remains to be elucidated about the mechanism for extracellular 4HNE-Trx1 adduct to regulate airway epithelial response during AECOPD. The newly identified proteins *via* proteomic analysis in this study may be an important clue to reveal novel mechanism of airway damage or remodeling in AECOPD patients, and moreover these proteins could be potential targets for pharmacological intervention in order to prevent and/or to treat AECOPD.

## Supporting information

S1 Table Differentially expressed proteins (≥ 5-fold change in the cells treated with 4HNE-Trx1 *vs* the cells treated with Trx1). (DOCX)

S1 Raw images (PDF)
