# Supplementary material for: Elevated serum 4HNE plus decreased serum thioredoxin: Unique feature and implications for acute exacerbation of chronic obstructive pulmonary disease
Source: PLoS One. 2021 Jan 25;16(1):e0245810. doi: 10.1371/journal.pone.0245810 (PMC7833214; doi:10.1371/journal.pone.0245810)
Supplement: S1 Table — (DOCX) [file pone.0245810.s001.docx]

**S1 Table. Differentially expressed proteins (≥ 5-fold change in the cells treated with 4HNE-Trx1 *vs* the cells treated with Trx1)**

| Classification | GENE | Description | UniProt ID | Function | FC^a^ |
| --- | --- | --- | --- | --- | --- |
| RNA metabolism (21)^b^ | UPF3B | Regulator of nonsense transcripts 3B | Q9BZI7 | Nonsense-mediated mRNA decay | -8.2 |
|  | RBM34 | RNA-binding protein 34 | P42696 | Alternative splicing | -8.4 |
|  | RBM12B | RNA-binding protein 12B | Q8IXT5 |  | 15.1 |
|  | RBMXL1 | RNA binding motif protein, X-linked-like-1 | Q96E39 | Pre-mRNA splicing | 5.0 |
|  | NOB1 | RNA-binding protein NOB1 | Q9ULX3 | mRNA degradation | 4.7 |
|  | CCDC137 | Coiled-coil domain-containing protein 137 | Q6PK04 | Ribosomal small subunit assembly | 8.2 |
|  | RPP25 | Ribonuclease P protein subunit p25 | Q9BUL9 | DNA damage response? | 4.8 |
|  | RRP7A | Ribosomal RNA-processing protein 7 homolog A | Q9Y3A4 | Ribosomal small subunit assembly | 6.1 |
|  | MRPS35 | 28S ribosomal protein S35, mitochondrial | P82673 | Structural constituent of ribosome/ mitochondrial translational | 5.2 |
|  | SYMPK | Symplekin | Q92797 | mRNA processing | 5.0 |
|  | NUP50 | Nuclear pore complex protein Nup50 | Q9UKX7 | mRNA transport/DNA damage response | 5.6 |
|  | CPSF3 | Cleavage and polyadenylation specificity factor subunit 3 | Q9UKF6 | 5'-3' exonuclease activity | -10.5 |
|  | DDX23 | Probable ATP-dependent RNA helicase DDX23 | Q9BUQ8 | Pre-mRNA splicing | -5.5 |
|  | DDX41 | Probable ATP-dependent RNA helicase DDX41 | Q9UJV9 | Pre-mRNA splicing | -7.3 |
|  | CELF1 | CUGBP Elav-like family member 1 | Q92879 | Pre-mRNA alternative splicing, mRNA translation and stability | -5.4 |
|  | ZNF326 | DBIRD complex subunit ZNF326 | Q5BKZ1 | Alternative splicing | -4.6 |
|  | ASH2L | Set1/Ash2 histone methyltransferase complex subunit ASH2 | Q9UBL3 | Alternative splicing / H3K4 methylation | 4.4 |
|  | ZMYND8 | Protein kinase C-binding protein 1 | Q9ULU4 | DNA damage response | 5.6 |
|  | PPP1R8 | Nuclear inhibitor of protein phosphatase 1 | Q12972 | Splicing factor/ phosphatase regulator/ repair DNA | -6.5 |
|  | TCEAL4 | Transcription elongation factor A protein-like 4 | Q96EI5 | Transcriptional regulation | -5.7 |
|  | GTF3C1 | General transcription factor 3C polypeptide 1 | Q12789 | Ribosome biogenesis /protein synthesis | 4.7 |
| immune homeostasis (5)^b^ | GTF3C5 | General transcription factor 3C polypeptide 5 | Q9Y5Q8 | Inflammatory response | 5.6 |
|  | PRKRA | Interferon-inducible double-stranded RNA-dependent protein kinase activator A | O75569 | Inhibition of translation and Induction of apoptosis | -5.6 |
|  | CCDC22 | Coiled-coil domain-containing protein 22 | O60826 | Regulation of NF-kappa-B signaling | 7.1 |
|  | CCDC86 | Coiled-coil domain-containing protein 86 |  | Immune homeostasis | -4.8 |
|  | NR3C | Nuclear receptor 3C |  | Steroid biogenesis and action | 5.6 |
| DNA process (11)^b^ | NCAPD3 | Condensin-2 complex subunit D3 | P42695 | DNA condensation | 6.1 |
|  | WRNIP1 | ATPase WRNIP1 | Q96S55 | DNA synthesis involved in DNA repair | 7.1 |
|  | HMGN4 | High mobility group nucleosome-binding domain-containing protein 4 | O00479 | Nucleosomal DNA binding | 4.8 |
|  | HMGN5 | High mobility group nucleosome-binding domain-containing protein 5 | P82970 | Chromatin organization, Transcription regulation | 4.8 |
|  | HMGN2 | Non-histone chromosomal protein HMG-17 | P05204 | Immune response/antitumor | 10.6 |
|  | BRD4 | Bromodomain-containing protein 4 | O60885 | Chromatin regulator， DNA damage | 12.3 |
|  | GTF2H1 | General transcription factor IIH subunit 1 | P32780 | DNA repair | 7.4 |
|  | TYMS | Thymidylate synthase | P04818 | dTTP biosynthesis | -4.7 |
|  | DCTPP1 | dCTP pyrophosphatase 1 | Q9H773 | dCTP catabolic process | -5.9 |
|  | POLA2 | DNA polymerase alpha subunit B | Q14181 | DNA replication | -4.7 |
|  | NUSAP1 | Nucleolar and spindle-associated protein 1 | Q9BXS6 | Cell cycle, Cell division, Mitosis | -5.4 |
| Transport (6)^b^ | RAB5A | Ras-related protein Rab-5A | P20339 | Transport | 10.4 |
|  | VAMP8 | Vesicle-associated membrane protein 8 | Q9BV40 | Transport | 8.8 |
|  | FTH1 | Ferritin heavy chain | P02794 | Cellular iron ion homeostasis | -7.6 |
|  | TMED5 | Transmembrane emp24 domain-containing protein 5 | Q9Y3A6 | Transport | 4.7 |
|  | JAGN1 | Protein jagunal homolog 1 | Q8N5M9 | Vesicle-mediated transport | 5.6 |
|  | ARFIP1 | Arfaptin-1 | P53367 | Intracellular protein transport | -4.7 |
| Cytoskeleton (9)^b^ | ITGA1 | Integrin alpha-1 | P56199 | Cell adhesion | 4.5 |
|  | TPM2 | Tropomyosin beta chain | P07951 | Actin filament organization | -6.4 |
|  | MYO5C | Unconventional myosin-Vc | Q9NQX4 | Motor activity | 26.0 |
|  | FLOT2 | Flotillin-2 | Q14254 | Epidermal cell adhesion | 6.6 |
|  | KRT2 | Keratin, type II cytoskeletal 2 epidermal | P35908 | Structural constituent of epidermis | -49.3 |
|  | KRT10 | Keratin, type I cytoskeletal 10 | P13645 | Establishment of the epidermal barrier | -7.9 |
|  | KRT9 | Keratin, type I cytoskeletal 9 | P35527 | Structural constituent of cytoskeleton | -41.9 |
|  | CLASP1 | CLIP-associating protein 1 | Q7Z460 | Microtubule cytoskeleton organization | 4.5 |
|  | TUBB6 | Tubulin beta-6 chain | Q9BUF5 | Structural constituent of cytoskeleton | -7.3 |
| Cell process (6)^b^ | OGFOD1 | Prolyl 3-hydroxylase OGFOD1 | Q8N543 | Regulating protein translation termination efficiency | -9.9 |
|  | RALB | Ras-related protein Ral-B | P11234 | Apoptosis, Cell cycle, Cell division | 8.8 |
|  | PCNP | PEST proteolytic signal-containing nuclear protein | Q8WW12 | Cell cycle regulation | -4.6 |
|  | HDGFRP2 | Hepatoma-derived growth factor-related protein 2 | Q7Z4V5 | Regulation of cyclin D1 expression | 5.5 |
|  | BZW2 | Basic leucine zipper and W2 domain-containing protein 2 | Q9Y6E2 | Cell differentiation | -6.7 |
|  | PRRC2C | Protein PRRC2C | Q9Y520 | Hematopoietic progenitor cell differentiation | -18.1 |
| Protein process (9)^b^ | FKBP9 | Peptidyl-prolyl cis-trans isomerase FKBP9 | P68106 | Protein folding | 6.5 |
|  | SRPRB | Signal recognition particle receptor subunit beta | Q9Y5M8 | IRE1-mediated unfolded protein response | 6.3 |
|  | GNG12 | Guanine nucleotide-binding protein G(I)/G(S)/G(O) subunit gamma-12 | Q9UBI6 | Signal transduction | 7.5 |
|  | HTRA1 | Serine protease HTRA1 | Q92743 | Serine protease | 8.7 |
|  | KIAA0564 | Von Willebrand factor A domain-containing protein 8 | A3KMH1 | ATPase activity | 6.8 |
|  | LRPAP1 | Alpha-2-macroglobulin receptor-associated protein | P30533 | Molecular chaperone for LDL receptor-related proteins | 5.3 |
|  | MRPS35 | 28S ribosomal protein S28, mitochondrial | Q9Y2Q9 | Mitochondrial translation regulation | 4.7 |
|  | NPR3 | Atrial natriuretic peptide receptor 3 | P17342 |  | 6.0 |
|  | CD99 | CD99 antigen | P14209 | Regulation of immune response | 7.4 |
| Metabolism (11)^b^ | MGEA5 | Protein O-GlcNAcase | O60502 | Glycoprotein catabolic process | -4.6 |
|  | GBE1 | 1,4-alpha-glucan-branching enzyme | Q04446 | Glycogen biosynthetic process | -12.9 |
|  | GNPDA1 | Glucosamine-6-phosphate isomerase 1 | P46926 | Glucosamine catabolic process | -4.9 |
|  | PAFAH1B3 | Platelet-activating factor acetylhydrolase IB subunit gamma | Q15102 | Lipid catabolic process | -8.6 |
|  | ERLIN2 | Erlin-2 | O94905 | Cholesterol metabolic process | 4.5 |
|  | LBR | Delta(14)-sterol reductase | Q14739 | Cholesterol biosynthesis | 6.0 |
|  | PLA2G4A | Cytosolic phospholipase A2 | P47712 | Arachidonic acid metabolic process、Lipid metabolism | -5.1 |
|  | PIGS | GPI transamidase component PIG-S | Q96S52 | GPI-anchor biosynthesis | -5.9 |
|  | RCN2 | Reticulocalbin-2 | Q14257 |  | -6.2 |
|  | UBE2K | Ubiquitin-conjugating enzyme E2 K | P61086 | Ubiquitin-dependent protein catabolic process | -4.9 |
|  | HIBCH | 3-hydroxyisobutyryl-CoA hydrolase, mitochondrial | Q6NVY1 | Branched-chain amino acid catabolism | -14.2 |
| Electron transport (4)^b^ | NDUFS7 | NADH dehydrogenase [ubiquinone] iron-sulfur protein 7, mitochondrial | O75251 | Respiratory chain | 5.5 |
|  | OXA1L | Mitochondrial inner membrane protein OXA1L | Q15070 | Assembly of cytochrome oxidase | 5.5 |
|  | CYB5R2 | NADH-cytochrome b5 reductase 2 | Q6BCY4 |  | -7.4 |
|  | ME2 | NAD-dependent malic enzyme, mitochondrial | P23368 | Tricarboxylic acid cycle | 5.4 |
| Others (10)^b^ | ADSL | Adenylosuccinate lyase | P30566 | Purine biosynthesis | -8.5 |
|  | MTAP | S-methyl-5'-thioadenosine phosphorylase | Q13126 | L-methionine biosynthesis via salvage pathway | -4.8 |
|  | CDKN2A | Cyclin-dependent kinase inhibitor 2A | P42771 | Cell cycle negative regulator of the proliferation | -11.3 |
|  | NCOA7 | Nuclear receptor coactivator 7 | Q8NI08 | Oxidation resistance gene | -7.3 |
|  | NUBP2 | Cytosolic Fe-S cluster assembly factor NUBP2 | Q9Y5Y2 | Cilium biogenesis/degradation | -7.8 |
|  |  |  |  |  |  |
|  | AAMP | Angio-associated migratory cell protein | Q13685 | Angiogenesis and cell migration | -5.3 |
|  | DDI2 | Protein DDI1 homolog 2 | Q5TDH0 | Aspartic protease | -5.9 |
|  | FSTL1 | Follistatin-related protein 1 | Q12841 | Binds heparin | -5.9 |
|  | TMEM33 | Transmembrane protein 33 | P57088 | Regulator of the tubular endoplasmic reticulum (ER) network | 14.5 |
|  | AUP1 | Ancient ubiquitous protein 1 | Q9Y679 |  | 5.1 |

^a^ Fold change was calculated using 4HNE-Trx1/Trx1 Area Ratio. ^b^ Number of proteins.
