# Supplementary material for: Elevated serum 4HNE plus decreased serum thioredoxin: Unique feature and implications for acute exacerbation of chronic obstructive pulmonary disease
Source: PLoS One. 2021 Jan 25;16(1):e0245810. doi: 10.1371/journal.pone.0245810 (PMC7833214; doi:10.1371/journal.pone.0245810)

Fig.1C

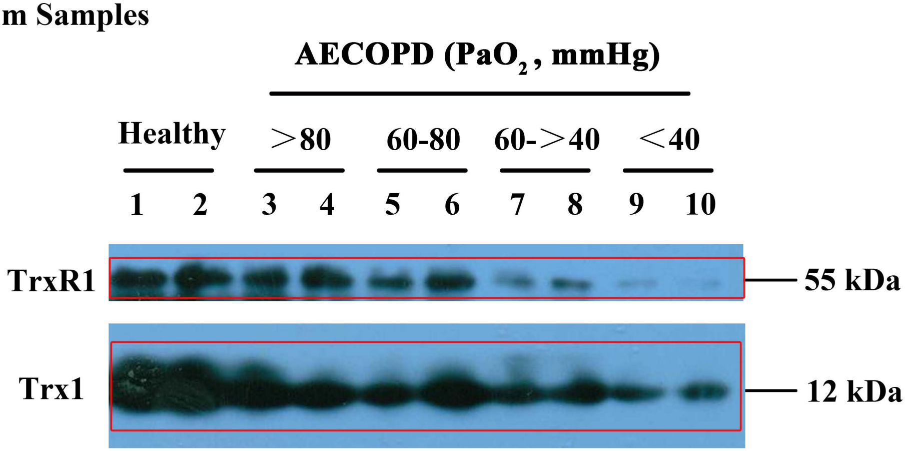
Serum Samples
AECOPD (PaO_2, mmHg)
Healthy
>80
60-80
60->40
<40
1 2 3 4 5 6 7 8 9 10
TrxR1
55 kDa
Trx1
12 kDa

Fig.1E

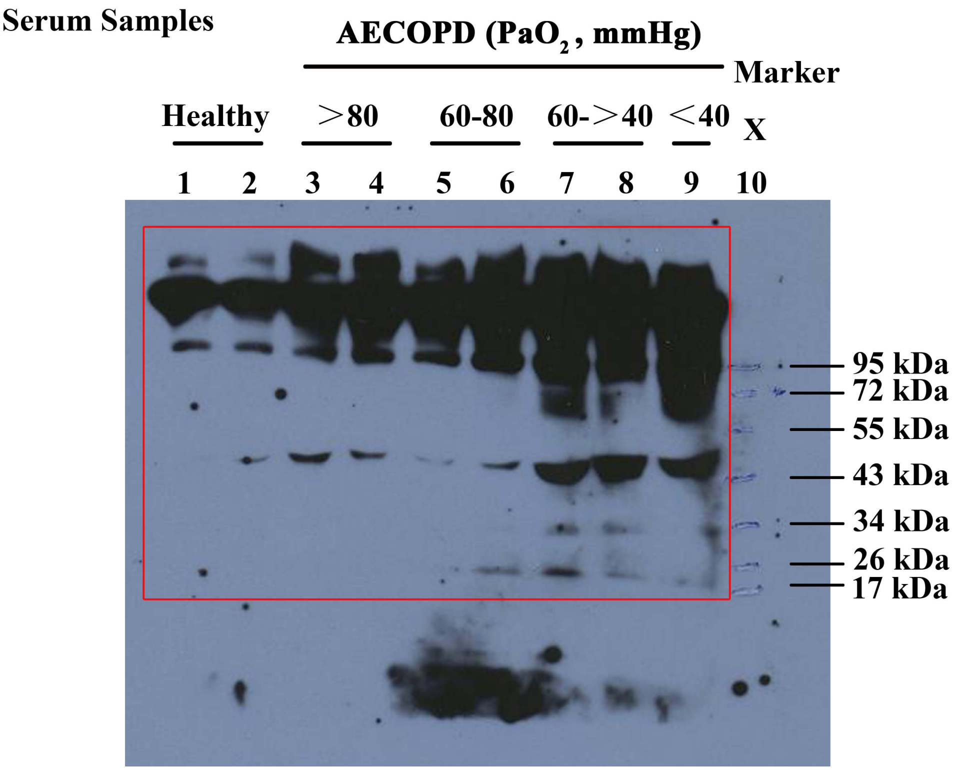
Serum Samples
AECOPD (PaO_2, mmHg)
Marker
Healthy
>80
60-80
60->40
<40
X
1 2 3 4 5 6 7 8 9 10
95 kDa
72 kDa
55 kDa
43 kDa
34 kDa
26 kDa
17 kDa

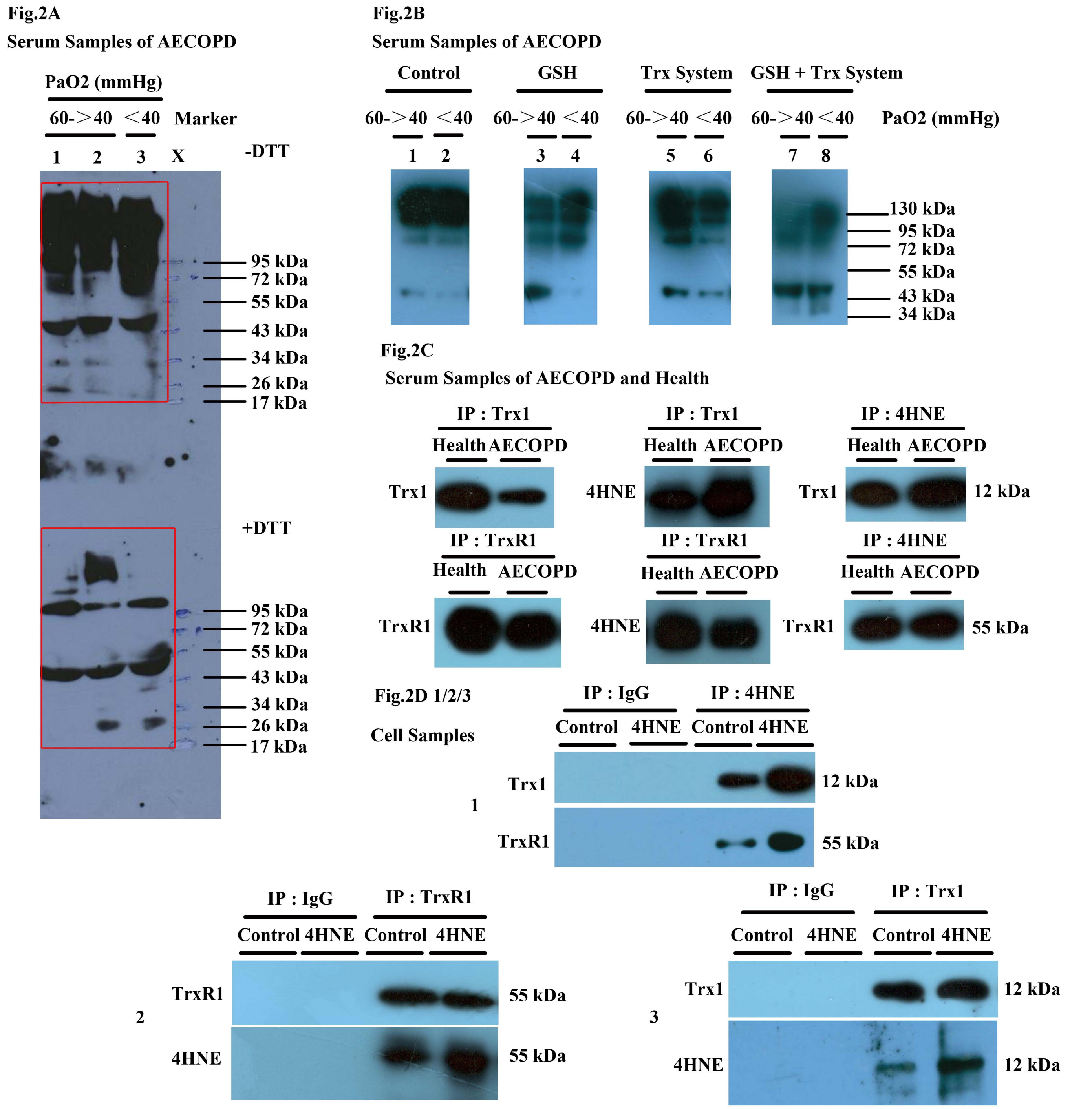

Fig.2A
Serum Samples of AECOPD
PaO2 (mmHg)
60->40 <40 Marker
1 2 3 X
-DTT
95 kDa
72 kDa
55 kDa
43 kDa
34 kDa
26 kDa
17 kDa
+DTT
95 kDa
72 kDa
55 kDa
43 kDa
34 kDa
26 kDa
17 kDa
Fig.2B
Serum Samples of AECOPD
Control
GSH
Trx System
GSH + Trx System
60->40 <40 60->40 <40 60->40 <40 60->40 <40 PaO2 (mmHg)
1 2 3 4 5 6 7 8
130 kDa
95 kDa
72 kDa
55 kDa
43 kDa
34 kDa
Fig.2C
Serum Samples of AECOPD and Health
IP : Trx1
Health AECOPD
Trx1
IP : Trx1
Health AECOPD
4HNE
IP : 4HNE
Health AECOPD
Trx1
12 kDa
IP : TrxR1
Health AECOPD
TrxR1
IP : TrxR1
Health AECOPD
4HNE
IP : 4HNE
Health AECOPD
TrxR1
55 kDa
Fig.2D 1/2/3
Cell Samples
IP : IgG
IP : 4HNE
Control 4HNE Control 4HNE
Trx1
12 kDa
1
TrxR1
55 kDa
IP : IgG
IP : TrxR1
Control 4HNE Control 4HNE
TrxR1
55 kDa
2
4HNE
55 kDa
IP : IgG
IP : Trx1
Control 4HNE Control 4HNE
Trx1
12 kDa
3
4HNE
12 kDa

Fig. 3E

Cell Samples

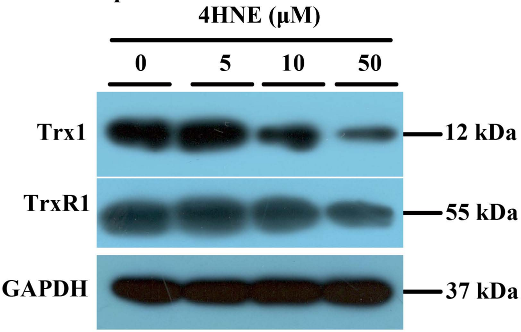

Fig. 3F

Cell Samples

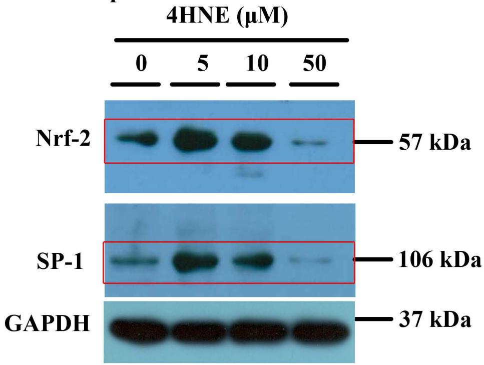

Fig. 3G

Cell Samples

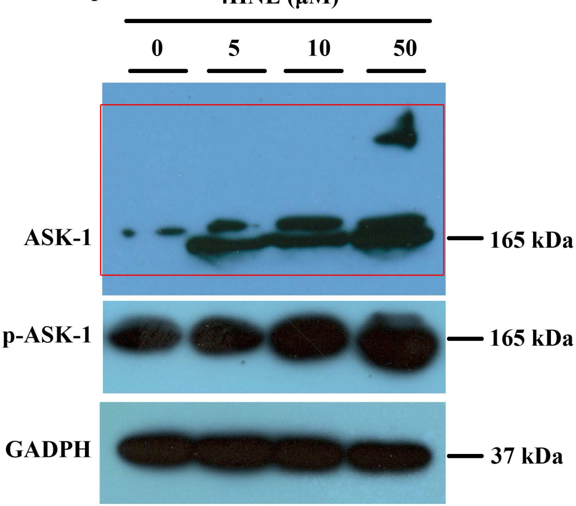

Fig. 5A

Cell Samples

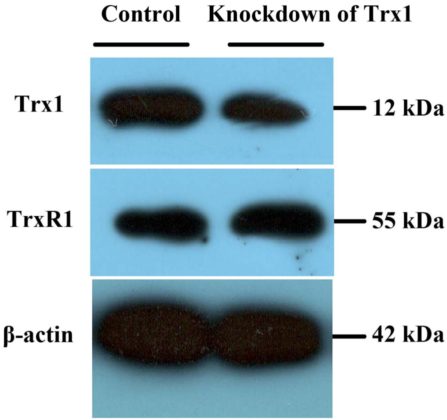

Supplement: S1 Raw images — (PDF) [file pone.0245810.s002.pdf]
